# Diagnosis and treatment of neurogenic dysphagia – S1 guideline of the German Society of Neurology

**DOI:** 10.1186/s42466-021-00122-3

**Published:** 2021-05-04

**Authors:** Rainer Dziewas, Hans-Dieter Allescher, Ilia Aroyo, Gudrun Bartolome, Ulrike Beilenhoff, Jörg Bohlender, Helga Breitbach-Snowdon, Klemens Fheodoroff, Jörg Glahn, Hans-Jürgen Heppner, Karl Hörmann, Christian Ledl, Christoph Lücking, Peter Pokieser, Joerg C. Schefold, Heidrun Schröter-Morasch, Kathi Schweikert, Roland Sparing, Michaela Trapl-Grundschober, Claus Wallesch, Tobias Warnecke, Cornelius J. Werner, Johannes Weßling, Rainer Wirth, Christina Pflug

**Affiliations:** 1 Klinik für Neurologie, Universitätsklinik Münster, 48149 Münster, Germany; 2 Klinik für Neurologie und Neurologische Frührehabilitation, Klinikum Osnabrück, Am Finkenhügel 1, 49076 Osnabrück, Germany; 3 Zentrum für Innere Medizin, Klinikum Garmisch-Partenkirchen GmbH, Auenstraße 6, 82467 Garmisch-Partenkirchen, Germany; 4 Klinik für Neurologie und Neurointensivmedizin, Klinikum Darmstadt, Grafenstr. 9, 64283 Darmstadt, Germany; 5 Raiffeisenstr. 9c, 85716 Unterschleißheim, Germany; 6 Ferdinand-Sauerbruch Weg 16, 89075 Ulm, Germany; 7 Universitätsspital Zürich, ORL-Klinik, Abteilung für Phoniatrie und Klinische Logopädie, Frauenklinikstr. 24, 8091 Zürich, Schweiz; 8 Schule für Logopädie, Universitätsklinikum Münster, Kardinal-von-Galen-Ring 10, 48149 Münster, Germany; 9 KABEG Gailtal-Klinik, Radniger Straße 12, 9620 Hermagor, Österreich; 10 Universitätsklinik für Neurologie und Neurogeriatrie, Johannes Wesling Klinikum Minden, Hans-Nolte Strasse 1, 32429 Minden, Germany; 11 Private Universität Witten/Herdecke gGmbH, Alfred-Herrhausen-Straße 50, 58448 Witten, Germany; 12 University Medical Centre Mannheim, Theodor-Kutzer-Ufer 1-3, 68167 Mannheim, Germany; 13 Abteilung Sprach-, Sprech- und Schlucktherapie, Schön Klinik Bad Aibling SE & Co. KG, Kolbermoorer Str. 72, 83043 Bad Aibling, Germany; 14 Schön Klinik München Schwabing, Parzivalplatz 4, 80804 München, Germany; 15 Medizinische Universität Wien, Teaching Center / Unified Patient Program, AKH Wien, Währinger Gürtel 18-20, 1090 Wien, Österreich; 16 Universitätsklinik für Intensivmedizin, Inselspital, Universitätsspital Bern, 3010 Bern, Schweiz; 17 Schinkelstraße 9, München, Germany; 18 REHAB Basel, Klinik für Neurorehabilitation und Paraplegiologie, Im Burgfelderhof 40, 4012 Basel, Schweiz; 19 VAMED Klinik Hattingen GmbH, Rehabilitationszentrum für Neurologie, Neurochirurgie, Neuropädiatrie, Am Hagen 20, 45527 Hattingen, Germany; 20 Klinische Abteilung für Neurologie, Therapeutischer Dienst, Universitätsklinikum Tulln, Karl Landsteiner Privatuniversität für Gesundheitswissenschaften, Alter Ziegelweg 10, 3430 Tulln an der Donau, Österreich; 21 BDH-Klinik Elzach gGmbH, Am Tannwald 1, 79215 Elzach, Germany; 22 Sektion Interdisziplinäre Geriatrie, Klinik für Neurologie, Medizinische Fakultät, Uniklinik RWTH Aachen, Pauwelsstraße 30, 52074 Aachen, Germany; 23 Zentrum für Radiologie, Neuroradiologie und Nuklearmedizin, Clemenskrankenhaus Münster, Düesbergweg 124, 48153 Münster, Germany; 24 Klinik für Altersmedizin und Frührehabilitation, Marien Hospital Herne, Universitätsklinikum der Ruhr-Universität Bochum, Katholische Kliniken Rhein-Ruhr, Hölkeskampring 40, 44625 Herne, Germany; 25 Klinik und Poliklinik für Hör-, Stimm- und Sprachheilkunde, Universitäres Dysphagiezentrum Hamburg, Universitätsklinikum Hamburg-Eppendorf, Martinistraße 52, 20246 Hamburg, Germany

## Abstract

**Introduction:**

Neurogenic dysphagia defines swallowing disorders caused by diseases of the central and peripheral nervous system, neuromuscular transmission, or muscles. Neurogenic dysphagia is one of the most common and at the same time most dangerous symptoms of many neurological diseases. Its most important sequelae include aspiration pneumonia, malnutrition and dehydration, and affected patients more often require long-term care and are exposed to an increased mortality. Based on a systematic pubmed research of related original papers, review articles, international guidelines and surveys about the diagnostics and treatment of neurogenic dysphagia, a consensus process was initiated, which included dysphagia experts from 27 medical societies.

**Recommendations:**

This guideline consists of 53 recommendations covering in its first part the whole diagnostic spectrum from the dysphagia specific medical history, initial dysphagia screening and clinical assessment, to more refined instrumental procedures, such as flexible endoscopic evaluation of swallowing, the videofluoroscopic swallowing study and high-resolution manometry. In addition, specific clinical scenarios are captured, among others the management of patients with nasogastric and tracheotomy tubes. The second part of this guideline is dedicated to the treatment of neurogenic dysphagia. Apart from dietary interventions and behavioral swallowing treatment, interventions to improve oral hygiene, pharmacological treatment options, different modalities of neurostimulation as well as minimally invasive and surgical therapies are dealt with.

**Conclusions:**

The diagnosis and treatment of neurogenic dysphagia is challenging and requires a joined effort of different medical professions. While the evidence supporting the implementation of dysphagia screening is rather convincing, further trials are needed to improve the quality of evidence for more refined methods of dysphagia diagnostics and, in particular, the different treatment options of neurogenic dysphagia. The present article is an abridged and translated version of the guideline recently published online (https://www.awmf.org/uploads/tx_szleitlinien/030-111l_Neurogene-Dysphagie_2020-05.pdf).

## Introduction

The present article is an abridged and translated version of the guideline recently published online (https://www.awmf.org/uploads/tx_szleitlinien/030-111l_Neurogene-Dysphagie_2020-05.pdf). The act of swallowing is a highly complex neuromuscular process that requires precise bilateral coordination of more than 25 muscle pairs. Using different imaging techniques, numerous physiological studies have consistently demonstrated that apart from the well-established role of the brain stem, different cortical areas are involved in the modulation of swallowing. Based on these findings, reorganization mechanisms have been further explored and form the neuroscientific basis for treatment approaches using different neurostimulation modalities.

Neurogenic dysphagia defines swallowing disorders caused by diseases of the CNS, PNS, neuromuscular transmission, or muscles. In contrast to this uniformity suggestive term, swallowing disorders caused by specific diseases differ considerably in terms of their clinical presentation, the respective therapeutic options, and the prognosis. Dysphagia is one of the most common and at the same time most dangerous symptoms of many neurological diseases. Impaired deglutition is initially found in at least 50% of all patients with ischemic or hemorrhagic stroke [1]. Affected patients have a 4 times increased risk of aspiration pneumonia, suffer more often from a long-lasting severe disability, are more often discharged to nursing homes, and also show significantly increased mortality [2]. Comparable numbers have been published for traumatic brain injury with a reported incidence of clinically relevant dysphagia in about 60% of patients [3]. In this patient collective, the presence of dysphagia is associated with a significantly extended time on mechanical ventilation and a longer need for artificial nutrition. In all Parkinson syndromes, neurogenic dysphagia is also a major risk factor for pneumonia, which is the leading cause of death in these patients [4, 5]. Furthermore, swallowing disorders in these patients are associated with a reduced quality of life, insufficient drug effects, and malnutrition [6, 7]. 20-30% of patients with dementia have severe dysphagia with silent aspiration that goes unnoticed by the patients [8]. Dysphagia is also a prominent clinical feature in various neuromuscular diseases. Up to 30% of patients with amyotrophic lateral sclerosis present with impaired swallowing at diagnosis [9] and practically all of them develop dysphagia as the disease progresses. Myasthenia Gravis manifests itself in 15% of cases with swallowing impairments. As the illness progresses, over 50 % of all patients are affected, and in more than 50 % of cases, a myasthenic crisis is preceded by dysphagia [10]. In multiple sclerosis, dysphagia occurs in more than one-third of patients and is linked to increased morbidity and mortality [11]. Patients with inflammatory muscle disorders are also often subject to swallowing impairment. The frequency is approximately 20 % in dermatomyositis, 30–60 % in polymyositis, and between 65 and 86 % in inclusion body myositis [12]. Finally, dysphagia is also a major diagnostic and therapeutic challenge in the intensive care unit [13]. Regardless of the primary illness, 70–80 % of patients requiring prolonged mechanical ventilation present, at least temporarily, with significant swallowing impairment and aspiration after successful weaning, probably due to a critical illness polyneuropathy and structural changes caused by the artificial airway like edema of the arytenoids [14]. This impairment not only necessitates prolonged artificial nutrition, but is also linked to serious complications, such as pneumonia and the necessity for reintubation and is in addition an independent predictor of increased mortality [13].

Regardless of the underlying diseases, the risk of developing a swallowing disorder increases significantly with age. Thus, dysphagia is found in 30-40% of independently living older people [15], while more than 50% of nursing home residents [16] and approximately 70% of all geriatric in-patients are affected by this disorder [17]. As with other patient groups, in geriatric patients dysphagia increases the risk of pneumonia and malnutrition [18] with the critical consequences of reduced physical and mental capabilities and, ultimately, increased frailty [19].

Finally swallowing disorders can also occur as a side effect of pharmacotherapy or at least be critically worsened [20]. First of all, both typical and atypical neuroleptics may cause dysphagia which may occur as either bradykinetic or dyskinetic form [21]. As shown in a recent systematic review, there is a dose-response relationship between the dosage of neuroleptic medication and the risk of pneumonia [22]. Also, treatment with benzodiazepine receptor agonists is associated with an increased risk of pneumonia, although the pathophysiological link with a possible drug-induced dysphagia for this group of substances is not clearly documented [20]. Finally, experimental studies have shown that intravenously injection of opiates is associated with an acute deterioration of pharyngeal swallowing function and an increased risk of aspiration [23]. However, the clinical significance of this finding is still unclear, since in recently extubated intensive care patients, for example, the occurrence of silent aspirations did not correlate with the cumulative opiate dose [24].

This guideline addresses general issues regarding diagnosis and treatment of neurogenic dysphagia. More disease-specific topics are covered in respective guideline chapters (diagnosis of acute cerebrovascular diseases; Idiopathic Parkinson syndrome, Diagnosis and therapy of Myasthenia Gravis and Lambert-Eaton syndrome, etc.). For specific questions regarding nutritional therapy and tube feeding, the S3 guideline “Clinical Nutrition in Neurology” of the German Society for Nutritional Medicine (DGEM) [25] and the guideline “Clinical nutrition in neurology” of the European Society for Clinical Nutrition and Metabolism (ESPEN) offer further information [26]. The topic of hypersalivation, which is often relevant in the treatment of dysphagia patients, is addressed in the S2k guideline of the German Society for Otolaryngology, Head and Neck Surgery which is summarized below [27].

## Methods of guideline development

This S1 level guideline (AWMF-registry number 030/111) is based on a systematic pubmed search. Where possible, the following sources were used: prospective randomized intervention studies, case-control studies, cohort studies, systematic meta-analysis, Cochrane reviews and guideline publications. In addition, the Cochrane Library was browsed for systematic reviews on the subject of dysphagia. Further references have been added as part of the review process by the guidelines committee. The following search terms were used for the literature search in pubmed (period 01.01.1990 to 30.06.2020).

Diagnostics: dysphagia OR swallowing disorder AND screening OR clinical swallow evaluation OR evaluation OR assessment OR fiberoptic endoscopic evaluation of swallowing OR flexible endoscopic evaluation of swallowing OR FEES OR videofluoroscopic swallowing study OR VFSS OR modified barium swallow OR MBS OR manometry OR ultrasound OR magnetic resonance imaging OR MRI OR computed tomography OR CT.

Therapy: dysphagia OR swallowing disorder AND behavioral intervention OR fluid thickening OR modification or modification OR nutrition OR nasogastric tube OR percutaneous endoscopic gastrostomy OR oral hygiene OR oral health OR neurostimulation OR neuromuscular electrical stimulation OR NMES OR transcranial direct current stimulation OR tdcs OR repetitive transcranial magnetic stimulation OR rtms OR pharyngeal electrical stimulation OR electrical pharyngeal stimulation OR PES OR pharmacological treatment OR capsaicin OR TRPV OR dopaminergic OR amantadine OR angiotensin-converting enzyme inhibitor OR ACE-inhibitor OR decannulation.

Consensus-building procedures. The guideline was first drafted by the two guideline coordinators after verbal agreement and informal consensus-finding of the parties involved in the preparation of the guidelines. The entire guideline group communicated via email to form the consensus subsequently. The recommendations were graded based on the available scientific evidence from “can” as lowest, through “should” to “must” as the highest recommendation strength.

This guideline has been adopted by the guidelines commission of the German Society of Neurology (DGN) and the other involved medical societies (see acknowledgment).

## Diagnostics

### Medical history

**Recommendation 1: Taking the medical history should focus on general aspects, dysphagia-specific topics and dysphagia-related complications.**

**Recommendation 2: The use of specific questionnaires is recommended in addition to the carefully guided history interview.**

A detailed medical history should be taken during the examiner’s initial contact with the patient. The examiner gets a general impression of the patient’s condition, vigilance and cognition, communication ability, and expected compliance during the further diagnostic workup. In addition to the patient’s awareness of the disorder (which is often diminished, for example, in the case of a pronounced oral and/or pharyngeal sensory deficit), these factors are important prognostic criteria and equally relevant for the assessment of the patient’s therapeutic capacity. If the patient cannot provide information himself or self-perception is limited, relatives are the most important source of information. It is also indispensable to review the medical records, especially regarding swallowing problems and results of previous diagnostic or therapeutic interventions. Information on the following points should be gathered in the structured interview:

- underlying disease
- comorbidities
- medications (especially neuroleptics, recent dose changes)
- onset and course of the disease
- current diet
- social status
- previous diagnostics
- previous treatments.

Subsequently, *dysphagia-specific issues* are clarified:

- changes in eating and drinking behavior
- avoidance of certain foods and consistencies
- difficulty taking medication
- time needed for a meal
- posture during eating
- difficulties with chewing
- food residues after swallowing in the oral cavity or throat
- feeling of “food sticks in the throat”
- globus sensation (either during eating or independently from food intake)
- voice change
- throat clearing, coughing or shortness of breath during the meal or shortly thereafter
- oral regurgitation of the bolus
- nasal regurgitation
- temporal dimension of symptom development (acute, subacute, chronic progressive, chronic recurrent)
- subjectively perceived localization of dysphagia (oral, pharyngeal, oesophageal)
- Relation to certain conditions such as physical or psychological stress, time of day

In addition, it is necessary to ask specifically about possible *complications* of dysphagia:

- occurrence of pneumonia, bronchopulmonary infections and infections of undetermined source
- dehydrations
- weight loss (always determine height, weight, BMI).

Standardized questionnaires can be used for systematically taking the medical history. For example, for TBI patients, “Anamnesebogen zur klinischen Erfassung von Schluckstörungen nach Hirnverletzung” is available [28]. In order to detect dysphagia symptoms more quickly, the EAT-10 (Eating Assessment Tool) was developed and validated for various patient groups, including amyotrophic lateral sclerosis (ALS), COPD and head and neck tumors [29]. Various validated questionnaires are available to assess quality of life impairments caused by the swallowing disorder. McHorney developed the Swallowing Quality of Life (SWAL-QOL) questionnaire [30]. The Swallowing Disturbance Questionnaire (SDQ) designed for use in Parkinson's patients has meanwhile been successfully tested in a cohort of patients with mixed dysphagia etiologies [31, 32].

### Aspiration screening

**Recommendation 3: Standardized aspiration screening should be performed in neurological patients.**

**Recommendation 4: The evaluation of a negative screening result should be made in the context of other clinical variables. If these indicate an increased risk of dysphagia, a further dysphagia assessment should be carried out in spite of the inconspicuous screening.**

**Recommendation 5: If neurological patients are admitted to hospital due to an acute neurological disease or due to an acute exacerbation of a pre-existing neurological disease, aspiration screening should be carried out as soon as possible and should therefore ideally be implemented in the initial diagnostic algorithm.**

**Recommendation 6: Water swallow tests and multi-consistency tests are available for aspiration screening. The choice of the optimal test procedure should be made taking into consideration other factors, such as patient characteristics and the availability of further dysphagia diagnostics.**

**Recommendation 7: Pulse oxymetry should not be used for aspiration screening.**

The aim of the aspiration screening is to quickly and reliably identify patients at risk of aspiration by simple means in order to initiate prophylactic measures and further diagnostics. Screening procedures should be designed in a way that they can be carried out after appropriate training by different health-care workers also without extensive previous dysphagia-specific training. Most of the published test protocols have been evaluated in stroke patients, but also in mixed patient cohorts, and are characterized by relatively high sensitivity (> 80%, partly >90%) and moderate specificity at best (usually <60%). In numerous reviews and meta-analyses, almost exclusively dedicated to stroke patients, the various screening tests have been evaluated and compared. However, because comparative studies are missing so far, the optimal test paradigm has not been determined yet [33, 34]. Methodologically, the screening methods can be differentiated into the following three categories, (i) water swallowing tests, (ii) multi consistency tests, (iii) swallow provocation test. The relevance of pulse oxymetry for detection of aspiration is considered to be low, despite its use in various test protocols , since studies have demonstrated that a decline in oxygen saturation of >3% was neither predictive nor sensitive for aspiration [35].

Over the past few decades, a variety of water swallowing tests has (WST) been published and validated mainly in stroke patients. These tests generally evaluate whether the patient can drink a defined amount of water without clinical signs of aspiration. The result of the WST is always binary; either the patient has clinical signs of aspiration necessitating NPO (nil per os) and subsequently more refined diagnostics, or the test is inconspicuous, after which oral intake is possible. In contrast to the WST, multi-consistency tests (such as the “Gugging Swallowing Screen” [36] or the Volume-Viscosity Test [37] evaluate besides liquids also other consistencies and therefore allow for a graded stepwise rating of swallowing impairment and usually add dietary recommendations to their risk assessments. The “Swallow Provocation Test” exclusively examines the involuntary swallowing reflex and thereby focusses on the pharyngeal phase of deglutition. This test may be an alternative in non-cooperative patients who cannot receive an oral bolus [38]. Despite the considerable methodological differences between WST and multi-consistency tests there are no comparative studies of these screening approaches available. In everyday clinical practice, the use of a WST is considered when the evaluation of swallowing safety has priority and a further assessment of the swallowing function (see below) is available in a very short period of time. A multi-consistency test on the other hand is preferred if, in addition to the safety of swallowing, swallowing efficiency is also to be assessed and a more differentiated swallowing assessment for determining the optimal oral diet is not available within a reasonable time frame. As an example for this reasoning, the volume viscosity test is used as central element within an algorithm for dysphagia management of geriatric patients that can be flexibly adapted to the availability of further diagnostic procedures [39]

Due to the inherent possibility of false-negative screening results, in patients with negative screening tests other clinical variables should be additionally considered. The DGEM guideline “Clinical Nutrition in Neurology” for example, recommends further dysphagia assessment in stroke patients with negative screening, if the patient presents with other predictors of a dysphagia such as a severe neurological deficit, severe dysarthria or aphasia or a severe facial palsy [25]. In addition, this aspect has to be considered in groups of patients with a high risk of silent aspiration, such as Parkinson's disease [4].

The fundamental impact of a simple aspiration screening in patients with neurogenic dysphagia has been studied in recent years, especially in the context of acute stroke. In several prospective observational studies, the implementation of an aspiration screening was associated with a reduction of infectious complications [40]. In a prospective, multicenter observational study Hinchey and colleagues demonstrated that institutions that had established a formal aspiration screening showed significantly lower pneumonia and mortality rates than those without such an algorithm [41]. A recently conducted pre-post comparison showed that the implementation of a nurse-based aspiration screening resulted in a 50% reduction of pneumonia rates in stroke survivors [42]. Finally, in a large, retrospective register study with more than 60,000 patients, performing an aspiration screening after stroke was found to be time-critical. Thus, the risk of developing pneumonia was linearly linked to the latency of screening and increased from just over 3% with prompt clinical examination to almost 4.5% when testing was done later than 24 hours [43]. A second, methodologically similar study also described this association between delayed performance of aspiration screening and increased risk of pneumonia [44].

### Dysphagia-Assessment

**Recommendation 8: The clinical swallowing examination should be based on validated protocols.**

**Recommendation 9: The dysphagia assessment should include a clinical swallowing examination and instrumental diagnostics, especially in the case of unclear patho-mechanism and/or unclear assessment of swallowing safety and swallowing efficacy.**

**Recommendation 10: FEES and VFSS are complementary methods of instrumental dysphagia assessment and should therefore, ideally, be both available.**

**Recommendation 11: FEES should preferably be used for bedside examinations in severely motor-impaired, bedridden or uncooperative patients.**

**Recommendation 12: FEES should preferably be used for the assessment of pharyngeal secretion management and for the assessment of laryngeal and pharyngeal sensitivity.**

**Recommendation 13: Pathological structural findings determined by FEES are to be demonstrated to a specialist (ENT or phoniatrician).**

**Recommendation 14: VFSS should be used preferably for the differentiated assessment of the pharyngeal and oesophageal phase of the swallowing, in particular in suspected disorders of the upper esophagus sphincter.**

**Recommendation 15: Manometry should be used as a complementary diagnostic tool to evaluate the function of the upper and lower oesophageal sphincter and in suspected esophageal motility disorders.**

**Recommendation 16: The evaluation of the swallowing act by sonography, MRI, CCT or EMG can be performed in the context of scientific studies and is not yet part of routine diagnostics.**

**Recommendation 17: In the context of dysphagia management, consistency-specific swallowing safety and swallowing efficacy should be determined by clinical and instrumental diagnostics using validated scores.**

**Recommendation 18: The clarification of an etiologically undetermined dysphagia requires an interdisciplinary diagnostic work-up in which, depending on the clinical constellation, neurologists, ENTs, phoniatricians, speech language pathologists, geriatricians, gastroenterologists, and radiologists should be involved.**

**Recommendation 19: In case of dysphagia of unclear origin, the phenomenological pattern of the swallowing impairment should be described as precisely as possible by means of clinical and instrumental investigations in order to obtain information about its etiology and to enable a targeted diagnostic work-up.**

**Recommendation 20: In addition to assessing the swallowing of different food consistencies and quantities, in dysphagia patients in need of oral medication, pill swallowing should be routinely evaluated as part of instrumental diagnostics and the individually optimal formulation should be identified.**

**Recommendation 21: In general, an inserted nasogastric tube does not affect the swallowing act and should, therefore, not be removed by default for dysphagia diagnostics and related treatment.**

**Recommendation 22: Patients with a tracheal cannula should be managed by a multi-professional team.**

**Recommendation 23: In tracheotomized patients with the therapeutic aim of decannulation, swallowing function, oropharyngeal secretion management, vigilance and ability to cooperate, respiratory function, and airway anatomy, the voluntary and reflexive cough, as well as the amount, nature and clearing of the bronchial secretion, should be evaluated regularly.**

**Recommendation 24: In tracheotomized patients, swallowing function should be evaluated with FEES and in particular the parameters “secretion management”, “spontaneous swallowing rate”, and “laryngeal sensitivity” should be investigated.**

**Recommendation 25: In tracheotomized patients, the location, fit and patency of the cannula, presence of granulation tissue and the placement of any existing fenestration should be checked regularly.**

**Recommendation 26: If patients are intended to be weaned gradually from the tracheal cannula, a physiological air flow through the upper airway should be established to improve pharyngo-laryngeal sensitivity. Therefore, if possible in the clinical context, the tracheal cannula’s cuff should be intermittently deflated and the cannula be capped or a one-way speaking valve used.**

**Recommendation 27: During gradual weaning of the tracheal cannula, if necessary in the clinical context, the diameter of the inner cannula should be downsized to reduce the airway resistance.**

**Recommendation 28: A definitive decannulation is usually possible if the cannula’s cuff can be continuously deflated with the cannula simultaneously being capped for 24-48 h without complications**

#### Clinical swallowing examination

The detailed clinical swallowing examination (CSE) falls within the domain of appropriately trained speech and language therapists (SLTs). In addition to the assessment of the aspiration risk, the CSE also provides as accurate an assessment of the severity and phenomenological pattern of the swallowing impairment as possible as a basis for further diagnostics, dietary recommendations, and treatment planning. After taking the medical history (see above) and testing the patient’s attention and ability to cooperate, oropharyngeal structures, including oral hygiene and dental status, the function of the caudal cranial nerves, secretion and saliva management, respiratory-swallow coordination, voluntary and reflexive cough, voice function and voice quality, laryngeal motility, oropharyngeal sensitivity, and spontaneous swallowing frequency are examined. Thereafter, swallowing tests with different consistencies, usually in the order of soft, liquid, and solid, are performed. In the case of pathological findings, swallowing maneuvers are applied to improve the safety and efficacy of the swallow [45]. Various protocols are available for systematic examination and documentation of findings, e.g. the Bogenhausen Dysphagia Score (BODS) [46] or the “Mann-Assessment of Swallowing Ability (MASA)” [47].

Despite its widespread use in everyday clinical practice, the validity of the CSE is limited [48]. Leder and colleagues, for example, found in a cohort of acute stroke patients that the CSE has a relatively good sensitivity of 86% for the determination of aspiration risk, but, due to a specificity of just 30%, does not allow for any reliable conclusion with regards to the presence of an undisturbed swallowing act [49]. In a study by McCullough et al., both the intra- and the interrater reliability of most of the parameters collected in the CSE were insufficient [50]. Rangarathnam and McCullough showed in a cohort of 60 patients with post-stroke dysphagia that the findings of the CSE matched those of VFSS only for laryngeal elevation while other parameters of swallowing physiology (e.g. oral transit, swallowing reflex latency, total duration of swallowing act) are not correctly assessed. Remarkably, in the same study, the dietary recommendations based on the two modalities were rather consistent [51]. These studies show on the one hand that compared to a WST, the CSE provides clinically relevant additional information, particularly regarding the assessment of the oral phase. On the other hand, inherent weaknesses of the CSE regarding the assessment of safety and efficacy of swallowing and in particular the pharyngeal phase have become obvious.

#### Flexible endoscopic evaluation swallowing (FEES)

Flexible Endoscopic Evaluation Swallowing (FEES) has been established nowadays in many German acute and rehabilitation clinics as diagnostic standard for the evaluation of swallowing. For example, a recent survey of German Stroke Units shows that the FEES is available here in more than 70% of the facilities, which corresponds to an increase of approximately 25% in the 5-year time frame [52, 53]. Consequently, in the meantime FEES has also been included in the catalogue of structural criteria for the DSG-Stroke Unit certification [54]. In addition, FEES was mentioned in a recent international survey (Management of Dysphagia on the ICU, MAD^Icu^) by more than 80% of neurointensivists as a regularly applied diagnostics for swallowing evaluation [55]. In a second survey carried out in the Netherlands 60% of intensivists reported to have access to FEES [56]. The fact that three major specialist societies (DGN, DSG and DGG) are now jointly running a training program to provide a formalized training in this examination technique, reflects the constantly growing recognition and importance of FEES [57, 58]. In addition, the DGPP and DGHNOKHC have created a training curriculum for the diagnostics and therapy of oropharyngeal dysphagia with FEES as a main subject [59]. During FEES, a flexible rhinolaryngoscope is inserted transnasally via the lower or middle nasal meatus into the pharynx. FEES provides a comprehensive picture of the pharyngeal phase of swallowing and enables the detection of indirect signs of impairment within the oral and oesophageal phases. The aims of FEES are, in particular, to identify pathological movement patterns, to assess the effectiveness and safety of swallowing, to determine suitable food consistencies and feeding strategies and to guide the use of therapeutic maneuvers for the individual patient. The standard FEES protocol consists of following steps (i) anatomical-physiological examination, (ii) swallowing without and with defined test boli, (iii) review of the effectiveness of therapeutic methods [60]. Various scales are available for the evaluation of the salient endoscopic findings (e.g. penetration aspiration scale according to Rosenbek [61], the Yale Residue Scale [62], Secretion Severity Scale [63]; Scale for Quantification of premature spillage [64]. Besides the standard FEES protocol specific examination protocols have been developed and validated for various clinical issues (FEES Tensilon-Test and Fatigable Swallowing Test for the detection and follow-up evaluation of a myasthenic dysphagia; FEES-L-Dopa-Test for evaluation of L-Dopa-sensitive dysphagia in patients with Parkinson's syndrome; FEDSS for the grading and management of stroke-related dysphagia; Decannulating algorithm to assess the feasibility of decannulation in tracheotomized intensive care patients [65]). In addition, structural abnormalities found within FEES, no matter whether they are pathophysiologically related to impaired deglutition or not, need to prompt a consultation by an otolaryngologist or phoniatrician. For example, redness, swelling and mucosal thickening in the posterior glottic area can indicate a gastro-oesophago-pharyngeal reflux, which untreated can lead to severe pulmonary infections in patients at risk of aspiration.

The FEES registry study analyzed side effects and clinical impact of FEES in everyday clinical practice in a prospective multricenter design [66]. 2401 patients were recruited in 23 hospitals between 2014 and 2017. The diagnostic spectrum included all relevant neurological diseases associated with dysphagia, in particular stroke, Parkinson's disease, critical illness polyneuropathy, motor neuron disease, dementia, myasthenia gravis, and myopathies. The first main result of the study was that FEES was performed safely regardless of the examiner's previous experience and was well tolerated by the patients. Secondly, the study showed in accordance with Braun et al. that FEES had a significant impact on dysphagia management [67]. Based on the results of FEES, more than 40% of patients were able to obtain a more liberal oral diet, while more than 10% required a more cautious nutritional approach. In the subgroup of tracheotomized patients (N=447), based on FEES decannulation was possible in more than 25% of cases. A retrospective study showed that after the implementation of bedside FEES service on a stroke unit, a significant reduction in pneumonia rates (12% to7%) could be achieved. In addition, patients were more likely to receive a regular diet at discharge, while the duration of a non-oral nutrition and the duration of hospital stay increased under the new regimen [68].

#### Video fluoroscopic evaluation of swallowing

The Videofluoroscopic Swallowing Study (VFSS), or the modern digital method (Digital Fluoroscopic Swallowing Study, DFSS), is a contrast based, radiological examination of the entire swallowing act including oral, pharyngeal, and oesophageal stages. VFSS is today usually performed according to the Logemann standard [69]. Here, the patient is examined in the lateral view with liquid boli of increasing volume up to consecutive cup swallowing. The patient is then given semisolid and lastly solid test boli. Finally, if required, the patient is examined in the anterior-posterior view, which is particularly appropriate to detect lateral asymmetries (e.g., unilateral residues in the case of a one-sided pharyngeal palsy). This examination step can be complemented by a “Valsalva maneuver” to depict hypotonic parts of the pharyngeal constrictors or, very rarely, pharyngoceles. VFSS distinguishes between dysphagia symptoms (e.g. aspiration, residues) and underlying pathomechanisms [70]. In addition, this technique offers apart from qualitative parameters also quantitative measures, such as the oral onset time, the oral transit time, the pharyngeal transit time, the anterior-superior movement of the hyoid, the duration and width of the velopharyngeals closure and the duration and width of the opening of the upper oesophagus sphincter. In a large number of studies on different patient cohorts, it has been shown that specific VFSS parameters, such as the latency of the laryngeal closure and the opening of the UES are associated with penetration and aspiration [71, 72]. In addition, these and other VFSS findings have been identified as indicators for the recovery of swallowing function after stroke [73] or for the responsivity of dysphagia to swallowing interventions [74]. Besides these specific parameters global VFSS-based dysphagia scores that provide a graduation of dysphagia severity have also been developed and validated. For example, the "Modified Barium Swallow Study Impairment Profiles (MBSimP©™) aggregates 17 single parameters of swallowing physiology to an overall score [75]. The MBSImP has now been successfully used in basic research [76] and has been adopted to characterize dysphagia in COPD patients [77]. The DIGEST (Dynamic Imaging Grade of Swallowing Toxicity), on the other hand, summarizes parameters of swallowing safety and swallowing efficiency in a 5-step score [78], which has been employed in patients with oculopharyngeal muscular dystrophy and amyotrophic lateral sclerosis [79, 80]. The PDVFS (Parkinson Disease VFSS Scale) was developed as a disease-specific score to predict the risk of aspiration pneumonia [81]. Highlighting the concrete benefits for the dysphagia management a retrospective study showed that in dysphagic stroke patients VFSS could be used to safely change the route of feeding from artificial enteral nutrition via an NG-tube to an oral diet [82]

#### FEES and VFSS in comparison

Among the instrumental methods VFSS and FEES add to each other in their significance and regarding their advantages and disadvantages. Depending on the clinical scenario, one or the other method can yield the larger diagnostic gain, so that neither of the two techniques is considered to be the only gold standard of the dysphagia diagnostics, but both methods are considered complementary. Methodologically, VFSS offers the advantage that the entire swallowing act, including the oral phase, pharyngeal constriction, epiglottic inversion, hyolaryngeal elevation, upper oesophageal sphincter function and the oesophageal phase is captured in high time resolution. Based on VFSS it is possible to detect and to comprehensively describe complex pathomechanisms of swallowing disorders affecting laryngo-pharyngeal and -oesophageal interactions. Apart from radiation exposure disadvantages of VFSS are the need for patient transport and the relatively high demands on the patient's ability to cooperate. FEES on the other hand, is methodologically restricted by the fact that it is focused on the pharyngeal phase of swallowing and is affected by the so-called white-out phenomenon. The practical merits of FEES in everyday routine are that it can be performed at the bedside, thus facilitating examination of severely motor-impaired, bedridden or uncooperative patients, that follow-up examinations can be performed at short notice and, if necessary, frequently; and that oropharyngeal secretion management and efficacy of cleaning mechanisms, such as coughing and throat clearing, can be assessed simply and directly. During recent years, several studies have shown that VFSS and FEES are comparable regarding the detection of swallow-specific main findings. Thus, a meta-analysis of 6 studies concluded that FEES detected penetrations/aspirations as well as residues somewhat more sensitively than VFSS while premature spillage has been diagnosed equally well by both methods [83]. In newer studies performing VFSS and FEES simultaneously in smaller patient cohorts, there was also a moderate superiority of FEES for the detection of residues, while the results were not consistent in terms of penetration and aspiration, but in the majority of patients both methods matched reasonably well [84, 85]. So far, only one prospective study recruiting a heterogeneous cohort of dysphagic outpatients (n=126) investigated whether dysphagia management recommendations based on FEES or VFSS resulted in better outcomes [86]. The patients were followed up for one year after initial instrumental evaluation. There were no significant differences in terms of pneumonia incidence and pneumonia free interval between the two diagnostic modalities. Only in the subgroup of chronic stroke patients (n=45) pneumonia rate was higher in patients managed with VFSS (29%) than with FEES (5%).

#### Manometry

Manometry, in particular high-resolution manometry (HRM), allows the endoluminal pressure conditions in the pharynx and oesophagus to be measured during the swallowing act. The method is particularly suitable to prove relaxation disorders of the UES and motility disorders of the oesophagus (achalasia, diffuse oesophagospasm). Oesophageal manometry can be carried out with standard tubes for which standard values have been established [87]. In particular, the following parameters can be assessed: resting pressure, opening of the upper and lower oesophageal sphincters as well as peristalsis, pressure and amplitudes of the tubular oesophagus. For gastroenterological disorders of the oesophagus, an assessment based on the Chicago Classification is common [88]. Only in recent years, HRM was used for the assessment of the oesophageal motility in patients with neurological diseases, especially Parkinson syndromes [89], inflammatory myopathies [90] and Morbus Huntington [91]. In neurology HRM is particularly important in patients with opening disorders of the UES, e.g., as a result of myopathies or strategic brainstem infarctions. Here, HRM is instrumental for the indication of interventions at the UES (myotomy, dilatation, botulinum toxin injection) and post-interventional follow-up [92]. In contrast to oesophageal manometry, for the less common pharyngeal HRM there are still no standard values, since location and diameter of catheters used vary significantly. In addition to the resting tonus of the UES, peak pressures and contraction times of the velopharynx and base of tongue, total swallowing time, speed of pharyngeal contraction wave as well as the length of the active pharyngeal segment can be determined [93]. Recently, pharyngeal HRM has been used in various neurological diseases, such as stroke [94], Parkinson's disease [95] and inflammatory and genetically determined myopathies [96] to describe the pattern of swallowing impairment. In addition, HRM has been compared with other methods of instrumental evaluation of swallowing, in particular VFSS and FEES and has been identified as a prognostic indicator. Despite its potential to complement FEES and VFSS, pharyngeal manometry has still not been successfully integrated into routine dysphagia diagnostics. A recent survey involving 206 speech therapists from the US revealed that only 3.5% of them have access to HRM, and only half of this small group actually uses pharyngeal manometry for further diagnostics in patients with dysfunctions of the upper oesophageal sphincter [97].

#### Further modalities of instrumental evaluation of swallowing

With electromyography (EMG) the activation pattern of the majority of muscles involved in the swallowing act can be analyzed. Depending on the target muscle surface or needle electrodes need to be used [98]. In the literature the examination of four specific muscle groups with surface electrodes is recommended: M. orbicularis ori and M. masseter for the oral phase, the suprahyoidal or submental muscles (M. digastricus, M. mylohyoideus, M. geniohyoideus) and the infrahyoidal muscles (M. thyrohyoideus, M. sternothyroideus) for the pharyngeal phase. Needle electrodes can also be used to record the activation of the cricopharyngeal muscle as part of the upper oesophageal sphincter [99]. In the clinical routine, EMG is primarily used within swallowing therapy as biofeedback for enhancing the training of compensatory swallowing maneuvers. Here, muscle activity is recorded via submentally positioned EMG surface electrodes and can be presented graphically or audible to the patient [100].

With sonography dynamics of the oral swallow and the morphometry of oropharyngeal muscles can be studied. With a suitably positioned sector transducer, oral bolus transport, tongue motor activity, suprahyoidal as well as hyoidal and laryngeal movements can be visualized in real time. Even details of the intrinsic tongue muscles can be anatomically differentiated with modern ultrasound probes [97].

Dynamic magnetic resonance imaging (MRI), in particular adopting “Turbo Fast Low Angle Shot (turbo-FLASH) Sequences” at higher field strengths (≥ 3 Tesla), provides a series of anatomical images in rapidly acquired consecutive slices [101]. The dynamic MRI of the swallowing act is a non-invasive procedure without exposure to radiation with a relatively short examination time in a range of a few minutes and, therefore, theoretically also applicable to children. It also allows a direct view on the deeper oropharyngeal muscles and soft tissue, multiplanar and in motion, and (depending on the section plane) provides a simultaneous view of the oral cavity, pharynx, and larynx and a tracking of the bolus transit during the swallowing act. The time resolution now approaches that of VFSS at around 25 images/s. Comparative studies with either VFSS or FEES showed a good agreement of the dynamic MRI with these two established gold standard methods [102]. The main limitations of this technique in the context of dysphagia diagnostics are, on the one hand, the flat positioning in MRI that is usually non-physiological for swallowing and may exacerbate the swallowing impairment and, on the other hand, the limited ability to intervene due to the little space in the scanner, particularly when examining patients at risk of aspiration.

For computed tomography scanning, further technical developments such as the 320-row multi-slice CT offer potential applications for swallowing diagnostics. Due to the thin slice thickness and the high temporal resolution of the acquired images, four-dimensional data sets can be reconstructed in good temporal and spatial resolution. Inamoto and colleagues were able to perform a CT-based kinematic analysis of the swallowing act from the oral to the early oesophageal phase for the first time by using a scanner that enables the examination in a half-sitting position [103]. In initial studies, this technique was used to optimize the quantification of pharyngeal residues [104], to describe age-dependent changes in swallowing physiology [105] and to assess the influence of bolus volume [106], bolus viscosity [107] and swallowing maneuvers on the swallowing act [108].

The differential indications of the described instrumental procedures are summarized in Table 1.

**Table 1:** Differential indication of instrumental dysphagia diagnostics for the evaluation of neurogenic dysphagia [65]

Methods of instrumental dysphagia evaluation	Indications
Endoscopy (FEES)	gold standard; particularly suitable for assessment of saliva accumulation and for sensory testing, preferred method in stroke units and neurological intensive care units
Videofluoroscopy (VFSS)	gold standard; evaluation of all swallowing phases; particularly suitable for assessing intra-deglutitive aspiration, hyolaryngeal elevation, epiglottic tilt, contact of the tongue-base to the back of the pharyngeal wall and impaired opening of the upper oesophageal sphincter
Manometry	Recording of timing and amplitude of the pharyngeal and oesophageal contraction and impaired opening of upper and lower oesophageal sphincter (in particular important before possible cricopharyngeal myotomy), oesophageal motility disorders
Electromyography (EMG)	biofeedback, otherwise mainly experimental procedure
Sonography	currently mainly experimental procedure
Magnetic resonance imaging (MRI)	currently mainly experimental procedure
Computer tomography (CT)	currently mainly experimental procedure

#### Algorithm for a structured assessment of patients with neurogenic dysphagia

The questions targeted by comprehensive dysphagia diagnostics depend on the specific clinical context. Basically, two scenarios can be differentiated here: dysphagia with an already determined etiology and dysphagia of unclear etiology.

##### Dysphagia with determined etiology

If patients with an etiologically classified, known dysphagia are examined, dysphagia diagnostics pursue the goal of determining the optimal dysphagia management for the patient in addition to the treatment of the underlying disease. The most important task in this context usually is to determine the safest and most convenient form of nutrition. In addition, it should be tested in particular whether the use of specific techniques (e.g., chin-tuck maneuvers, Mendelsohn maneuvers) can improve the swallowing function. In view of the above-described immanent methodological advantages, and because of the increasing availability, safety and diagnostic yield, FEES should be carried out as the first instrumental method to clarify these questions [109]. The FEES registry study showed that in a heterogeneous, neurological patient cohort, the patients’ diet needed to be adjusted based on the FEES results in more than 50% of the patients [66]. In a second study, FEES required a change of feeding strategy in two thirds of the patients [67]. In a third study recruiting a cohort of Parkinson patients, 18% of patients without subjective swallowing impairment required compensatory techniques and regular swallowing therapy due to the objective severity of their dysphagia determined by FEES. In 8% of the same subgroup, the swallowing disorder was so severe that tube feeding was required [4]. The key parameters helping to determine safety and efficacy of swallowing are the consistency-specific assessment of penetration and aspiration on the one hand and residues on the other [109]. Established scores such as the penetration-aspiration scale [61, 110] and the Yale residue scale [62, 111], both of which have been validated in German, should be used for a more precise and easy-to-communicate rating.

If there are questions beyond the definition of nutritional management that cannot be answered adequately with FEES alone (e.g. extent UES dysfunction, additional presence of oesophageal dysphagia), further instrumental procedures, in particular the VFSS and HRM, should be used.

##### Etiologically undetermined dysphagia

**Fig. 1:**
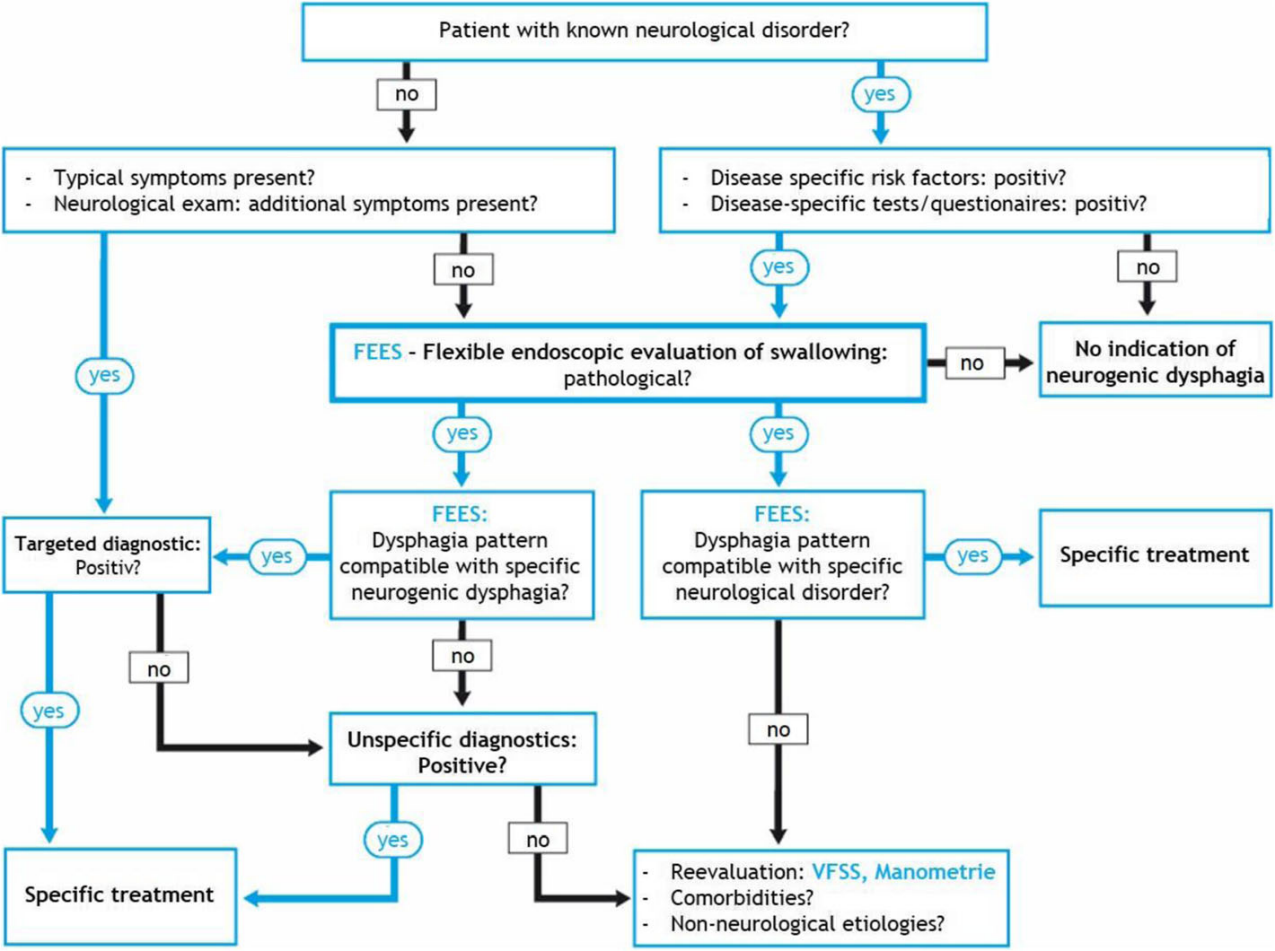
Structured algorithm for the diagnosis of neurogenic dysphagia [65]

Basically, the work-up in case of an etiologically unexplained dysphagia requires interdisciplinary diagnostics, which, depending on the clinical situation, should involve gastroenterologists, neurologists, otolaryngologists, phoniatricians, SLPs, geriatricians and radiologists. To differentiate between structural and neurogenic dysphagia, an appropriately qualified examination of the oropharynx and a pharyngolaryngoscopy are required. Further gastroenterological diagnostics using oesophagogastroscopy and manometry (see above) is indicated if there is a suspicion of oesophageal dysphagia. In order to initiate proper protective and rehabilitative measures, it is essential to diagnose the underlying dysphagia-causing disease. In addition, relevant statements regarding the prognosis can only be provided to patients and relatives if the etiology of neurogenic dysphagia has been clarified [65]. As shown in Fig. 1, the diagnostic procedure differs depending on whether a neurological disease is already known or not.

If no neurological diagnosis has previously been established, the further procedure depends on the presence of anamnestic or additional clinical symptoms that give rise to specific diagnostics (see Table 2). If, for example, the clinical neurological examination reveals cranial nerve paresis a cranial polyneuritis or a basal meningitis need to be considered. If a diagnosis can be made on the basis of this clinical information and the further examinations, a specific therapy can be initiated thereafter.

**Table 2:** Differential diagnosis of neurogenic dysphagia in relation to additional neurological symptoms (according to [65])

Additional neurological symptoms	Differential diagnoses
Acute CNS symptoms	cerebral infarctions/bleeding
	relapse of multiple sclerosis
Slowly progressive CNS symptoms	brain tumors
	chronic progressive multiple sclerosis
Brainstem symptoms	brainstem infarctions/bleeding
	multiple sclerosis
	listeria rhombencephalitis
	paraneoplastic brainstem encephalitis
Neurocognitive disorders	Alzheimer’s disease
	vascular dementia
	frontotemporal lobar degeneration
	Lewy body dementia
	progressive supranuclear palsy
Extrapyramidal motor symptoms	Parkinson’s disease
	Huntington’s disease
	Dystonias
	Neuroleptic-induced dysphagia
	Wilson’s disease
Progressive bulbar paralysis	Amyotrophic lateral sclerosis
	Pseudobulbar paralysis
	Primary lateral sclerosis
	Arnold–Chiari malformation, type I
	Kennedy’s disease
	Post-polio syndrome
	IgLON5 bulbar paralysis
Cerebellar symptoms	Multiple sclerosis
	Hereditary ataxias
	Niemann–Pick disease, type C
	Subacute cerebellar degeneration
Cranial nerve palsies	Skull base tumors
	Meningeosis neoplastica
	Basal meningitis
	Subtypes of Guillain–Barré syndrome
Ptosis and/or ocular symptoms	Subtypes of Guillain–Barré syndrome
	Myasthenia gravis
	Lambert–Eaton myasthenic syndrome
	Botulism
	Oculopharyngeal muscular dystrophy
	Mitochondrial myopathies
	Oculopharyngodistal myopathy
Neuropathy	Guillain–Barré syndrome
	Critical illness neuropathy
Myopathy	Myositis
	Myotonic dystrophies
	Duchenne muscular dystrophy
	Oculopharyngeal muscular dystrophy
	Mitochondrial myopathies
	Facioscapulohumeral muscular dystrophy
	Oculopharyngodistal myopathy
Myotonic syndrome	Myotonic dystrophies
Trismus and/or risus sardonicus	Tetanus

Instrumental diagnostics are used here primarily to plan dysphagia management and to evaluate the success of the targeted treatment (see above). However, if dysphagia is the sole or predominant symptom of a neurological disorder, differential diagnosis is often more difficult. In these cases, after a medical history and neurological examination, FEES should be carried out. In contrast to the situation outlined above, where the main aim of swallowing assessment was to propose a suitable dysphagia management strategy, in this scenario it is essential to carefully determine the phenotype of the swallowing impairment (Table 3) [65]. If there are specific or at least suggestive findings (e.g., disorder of the UES, fatigue of the swallowing muscles during the examination), the subsequent diagnostics should be focused on FEES-based differential diagnosis. In the case of an unspecific phenotype of swallowing impairment and depending on the clinical constellation, a contrast-enhanced MRI of the brain with thin sections of the brain stem, neurophysiological examinations, a FEES tensilon test, the determination of auto-antibody profiles, a lumbar puncture and / or a whole-body Muscle MRI should be considered next [65].

**Table 3:** Endoscopic phenotypes of neurogenic dysphagia [65]

Main findings	Neurological diseases	
	Peripheral	Central
I) Premature spillage	Early-stage ALS	Early-stage ALS, early-stage PSP, frontotemporal dementia, SPG7-HSP, acute stroke*
II) Delayed swallow reflex		Acute stroke*
III) Impaired pharyngeal bolus clearance (residue in valleculae >>> residue in piriform sinus)	Bulbospinal muscular atrophy, myotonic dystrophy type II, (critical illness neuropathy/myopathy,) early stage ALS	Early ALS, early-stage PD
IV) Impaired opening of upper oesophageal sphincter (residue in piriform sinus >>> residue in valleculae)	Inclusion body myositis (IBM)	Dorsolateral medulla oblongata infarction
V) Complex pathology (combination of I-IV, at least 2 equivalent patterns)	Severe myasthenia gravis, end-stage ALS, (GBS), myotonic dystrophy type I	End-stage ALS, advanced stages of PD and PSP
VI) Extrapyramidal motor impairment (one out of I-IV) plus movement disorder	–	Neuroleptic-induced dysphagia, PD, MSA, Huntington’s disease
VII) Fatigable oropharyngeal dysphagia (one out of I-IV plus swallowing fatigability)	Myasthenia gravis	(PD, ALS)

*All stroke locations apart from strokes confined to the dorsolateral medulla oblongata

#### Evaluation of pill swallowing

Taking oral medication, especially swallowing tablets, is a relevant problem for many patients with dysphagia. In addition to aspiration and the resulting complications and discontinuation of medication, unsuitable modification of the oral medication can often be observed (e.g., crushing, breaking, and opening of tablets and capsules), which may lead to numerous problems, such as decreased accuracy of dose, increased toxicity, reduced stability, and alteration of pharmacokinetics [112]. Therefore, especially in patients with dysphagia who are required to take oral medication, swallowing of tablets should be routinely evaluated as part of the swallowing assessment and the optimal formulation (if available) should be identified [20]. In a study recruiting Parkinson patients, almost 30% of them as well as about 15% of the control subjects showed impaired pharyngeal transfer of the placebo tablets [7]. A proof-of-principle study in an etiologically heterogeneous group of dysphagic patients (N = 36) also showed that an orodispersible tablet was easier to swallow than a conventional tablet of the same size [113].

#### Dysphagia assessment in patients with a nasogastric tube

In patients with severe neurogenic dysphagia, nasogastric tube feeding is often recommended, at least temporarily, to ensure safe and sufficient enteral feeding [26]. Despite contrary recommendations in other guidelines [114], from a practical point of view it is important to consider that a nasogastric tube should not be removed for instrumental or clinical dysphagia diagnosis or for dysphagia therapy. In several studies, each with a different design and patient cohort, no clinically relevant negative effects of the tube on the swallowing function and aspiration risk, both determined by FEES or VFSS, could be identified [115, 116]. However, it must be taken into account that a nasogastric tube can cause swelling of the arytenoids as well as lesions to the pharyngeal mucosa, which in turn may cause difficulty swallowing and may be an indication for the PEG placement.

#### Dysphagia assessment in tracheotomized patients

Tracheotomy, particularly the minimally invasive dilatation procedure, has become a standard procedure in most intensive care units, so that today the majority of long-term ventilated patients are ventilated via this airway access. After successful weaning from the respirator, the next therapeutic goal is to remove the tracheal cannula. In view of the high prevalence of swallowing disorders in tracheotomized patients, dysphagia assessment plays a major role in tracheal cannula management [117]. In principle, the following three methods are available.

1. The clinical swallow examination is usually carried out as the first diagnostic step in cannulated patients weaned from the respirator. After deflating the cannula’s cuff and careful subglottic suctioning a physiological air flow through the upper airway is achieved by capping the cannula or using a speaking valve. This is followed by the swallowing examination, which is based on the usual procedure, and in particular searches for clinical signs of penetration and aspiration of saliva and administered food boluses. In accordance with the low reliability of the clinical swallow examination for detecting these critical events the sensitivity of this method in comparison to gold standard FEES is low. Therefore, weaning a patient from the tracheal cannula cannot be controlled only based on the clinical swallow examination [118].
2. As a further clinical instrument, the Evans Blue Test (EBT) and the modified Evans Blue Test (mEBT, Evans blue dye test) have been introduced into practice [119]. To carry out the test, the cannula’s cuff is first deflated and subglottic and pharyngeal secretion is carefully suctioned. The patient then receives a few drops of food coloring directly on the tongue or the patient is given orally small amounts of food-color dyed liquid (EBT) and possibly other food consistencies (mEBT). After the swallow, subglottic suctioning is repeated. If colored secretion (EBT) or colored liquid (mEBT) are detected, a high risk of aspiration is suspected. According to several studies and a meta-analysis this method features an insufficient sensitivity [120], while only two studies with repeated suction tests suggest an acceptable accuracy of the (m) EBT [121, 122]. In summary, a negative (m) EBT is of no diagnostic value, but a positive (m) EBT is considered to be indicative of substantial risk of aspiration in tracheotomized patients. In view of this scientific context, the (m) EBT should be classified as a screening instrument that can be used to follow-up instrumental evaluation. The exclusive use of the (m) EBT to assess readiness for decannulation is not recommended.
3. Due to the limitations of the clinical procedures described above, FEES is also of relevance this context. Hafner and co-workers have already shown that based on FEES, almost 23% of 258 tracheotomized patients could be decannulated [123]. A comparable rate was also reported in the FEES registry study, where removal of the tracheal cannula was possible in 26.4% of the 447 tracheotomized patients [66]. In addition, Cohen et al. showed that decannulation immediately following the endoscopic evaluation was associated with fewer recannulations, a shorter period of non-assisted spontaneous breathing before decannulation, and a shorter hospital stay after decannulation than a more protracted decannulation management [124]. In order to increase the reliability of the endoscopic examination, a standardized procedure that focuses on the parameters management of secretions, spontaneous swallowing rate and laryngeal sensitivity has been developed [125]. The application of this algorithm in 100 tracheotomized intensive care patients weaned from the respirator allowed for a rapid and safe decannulation in more than half of this patient cohort; only in one case recannulation was necessary in the further course of treatment. It was also noteworthy that the clinical swallowing examination, which took into account the parameters vigilance, ability to cooperate, saliva swallowing, coughing as well as the amount of secretions suctioned from the tracheal cannula, would have recommended removal of the tracheal cannula in only half of these patients [125]. In the meantime, this decannulation algorithm has been successfully used as primary endpoint of a multicenter study [126] and was also recommended by a French guideline [127].

#### Further recommendations for tracheostomy tube management

Tracheotomized patients are usually treated by a multiprofessional team that, depending on the local conditions, is composed of intensive care physicians, otolaryngologists, phoniatricians, respiratory therapists, SLPs and specialized nursing staff [128]. Even if no prospective randomized trials have been done so far and recommendations therefore have only a weak evidence base [129], a large number of studies with different designs and also individual meta-analyzes suggest that this interdisciplinary approach improves the prerequisites for rapid and safe decannulation [128, 130, 131]. Frank and co-workers. demonstrated in a study with pre-post design that after the implementation of a multi-professional tracheal cannula management consistently high decannulation rates and a > 50% reduction of average cannulation times were achieved [130]. These results were confirmed in a meta-analysis of 7 other cohort studies with a comparable design [128], which, in addition to accelerated weaning from the tracheal cannula, also described a shortening of the length of stay on the intensive care unit and a reduction of complications subsequent to the implementation of multiprofessional teams. In addition to the swallowing function and oropharyngeal secretion management (see above), the dedicated decannulation assessment also evaluates the patient's alertness and ability to cooperate, the respiratory function and anatomy of the airway, the voluntary and reflectory cough as well as the amount, consistency and clearing of the bronchial secretion [132, 133]. Also, the position, fit and patency of the cannula, the presence of granulation tissue, and the position of the cannula’s fenestration, if any, should be checked by regular endoscopy [132]. Especially if gradual weaning from the tracheal cannula is required, recurrent and progressively longer trials of cuff deflation with simultaneous capping of the tracheal cannula or use of a speaking valve are used during the rehabilitation [132, 134]. On the one hand, this procedure helps patients to practice breathing through the upper airway. On the other hand, the restoration of the physiological air flow is likely to result in a restitution of the pharyngeal and laryngeal sensitivity with consecutively improved secretion management [135]. In a proof-of-principle study recruiting 20 tracheotomized stroke patients, Ledl and Ullrich showed that while capping of the tracheal cannula did not induce any changes in swallowing mechanics, swallowing safety improved resulting in lower Penetration-Aspiration-Scale scores [136]. Regular, temporary decannulation as part of swallowing diagnostics or therapy does not appear to have any advantage over temporary capping of the cannula. In methodically high-quality studies, there was no change in different parameters of swallowing mechanics and swallowing safety between the conditions “swallowing with unblocked and capped tracheal cannula” and “swallowing without tracheal cannula” [137, 138]. As an important intermediate step before starting longer periods of cuff deflation with capping or use of a speaking valve, downsizing the cannula’s inner diameter is frequently necessary to reduce airway resistance [139]. Definitive decannulation is usually possible if management of pharyngeal secretions is sufficient and patients stay respiratory stable and tolerate 24 to 48 hour periods of cuff deflation combined with capping without complications [132, 134].

## Therapy

A variety of different therapeutic methods are now available for the treatment of neurogenic dysphagia. Since the indication for a specific treatment is determined not only by the phenotype of swallowing impairment, but also by the underlying etiology of dysphagia, an appropriately focused diagnostic work-up (see above) is essential before the final therapeutic strategy is determined. In this section, dietary, behavioral, pharmacological, and local interventional treatment options as well as neurostimulation procedures are presented and the importance of oral hygiene in patients with dysphagia is explained.

### Dietary interventions

**Recommendation 29: Texture-modified diets, thickened liquids and / or systematic modifications of bolus size should only be prescribed based on the findings of a swallow examination.**

**Recommendation 30: Thickening of liquids can be used in patients with neurogenic dysphagia who show aspirations with liquids.**

**Recommendation 31: To improve patient compliance, different types of thickeners should be offered and tested.**

**Recommendation 32: Texture-modified diet can be used in patients with chronic dysphagia to improve their nutritional status.**

**Recommendation 33: Despite the use of texture-modified food and thickened liquids, patients with neurogenic dysphagia are at increased risk of malnutrition, dehydration, and aspiration pneumonia and should, therefore, be monitored for these complications.**

The use of texture-modified foods and thickened liquids has become one of the most common therapeutic strategies to address neurogenic dysphagia. The idea behind this approach arises from the assumption that modifying the properties of normal food and liquids will make them easier and safer to swallow [140]. Despite the widespread use of this intervention, its scientific foundation in many areas is still incomplete or not convincing.

For decades there were no established and universally used terminology and definitions to describe the target consistency recommended for patients with OD and to guide its preparation [140]. Only recently the “International Dysphagia Diet Standardisation Initiative” (IDDSI) has been established that pursues the goal to develop global standardized terminology and definitions for texture modified food and thickened liquids for individuals of all ages, in all care settings, and all cultures [141].

Despite this ongoing discussion focused on terminology and definition issues [142], the effect of liquid thickening on the safety and efficacy of the swallow has been extensively investigated [143]. The results of more than 30 studies were summarized and analyzed in two recent systematic reviews and a white paper [140, 144, 145]. Those papers unanimously conclude that thickening liquids reduces the risk of aspiration in different patient groups. Although the available data are insufficient to suggest particular viscosity values, the analysis put forward by Newman and colleagues suggest that on the continuum covering the whole spectrum from “thin”, “nectar”, “honey” to “spoon thick”, there seems to be a dose-response characteristic with thicker liquids being safer than thinner liquids [145]. As a flip side of the coin, liquid thickening seems to increase the risk of post-swallow residues [140, 144, 145]. Although not as unequivocal and as frequently studied as aspiration, several studies reported oral and/or pharyngeal residues with ultra-thick liquids [37, 72, 146, 147].

As an alternative or supplement to viscosity adaptation, the bolus volume can also be adjusted. As shown in a systematic review, bolus volumes ≤ 5 ml have a lower aspiration risk than bolus volumes ≥ 10 ml [148].

In addition to these effects focused on the physiology of swallowing, clinically relevant endpoints have also been studied in the context of liquid thickening and texture modifications. Contrasting with its positive effect on swallowing safety, liquid thickening has failed to substantially improve fluid intake in several studies [149, 150] and systematic reviews [151, 152]. The main reason for this is that thickened liquids are poorly tolerated due to changes in taste, a “coating feeling in the mouth” and an insufficient alleviation of thirst [153]. In addition to a compliance-related, reduced fluid intake, thickening of liquids, therefore, correlated with a reduced quality of life [154].

Apart from viscosity, thickening agents also influence other characteristics of the liquids, such as texture, taste, and appearance. There is first evidence that different types of thickeners, in particular starch and rubber-based products, differ in this respect, which can have an impact on patient compliance [147].

The impact of feeding strategies involving texture modified diets on oral intake has been assessed in one small RCT [155]. In elderly dysphagic nursing home residents both food intake and nutritional status were improved in the intervention group over a time period of 12 weeks. In addition, a cohort study recruiting acute stroke patients showed that by being given a dysphagia diet, patients could achieve more than 75% of their energy requirements [156].

The effect of dietary interventions to prevent aspiration pneumonia has been studied in several systematic reviews and Cochrane analyses related to patients with dementia [157, 158], geriatric stroke patients [159] and geriatric patients with oropharyngeal dysphagia of heterogeneous etiologies [151]. It is generally concluded that the number of high-quality studies is too low to recommend the use of texture modified food and thickened liquids for the prevention of aspiration pneumonia. Of particular relevance in this context is the large RCT by Robbins and co-workers, which included more than 500 patients with dysphagia due to Parkinson’s disease or dementia and proven aspiration of thin liquids in VFSS. This study did not find a significant difference in the incidence of aspiration pneumonia between the group receiving thickened liquids and the group being treated with chin-down posture and normal liquids [160].

While dietary interventions in isolation appear to have only a small effect size, this approach may be more effective and meaningful when used within a multidimensional concept to prevent aspiration pneumonia. Thus, the so-called „Minimal-Massive Intervention (MMI)", which aims to reach as many patients as possible with a resource-saving (minimal) intervention and thereby achieve a large ("massive") effect, comprises the components (i) fluid and food texture adaptation, (ii) dedicated oral care (see below) and (iii) nutritional supplementation and targets the group of frail geriatric patients [161]

In a prospective non-controlled intervention study, this set of measures was used to reduce mortality, pneumonia rates and the rate of re-hospitalization and to improve the nutritional status of patients compared to a historical control group [161].

### Behavioral swallowing interventions

**Recommendation 34: Before initiating behavioral swallowing interventions, the etiology and phenotype of dysphagia should be determined.**

**Recommendation 35: The Shaker maneuver should be used in patients with pharyngeal residues and impaired opening of the UES.**

**Recommendation 36: Expiratory muscle strength training (EMST) should be used to treat dysphagia in patients with motor neuron disease, stroke and M. Parkinson. EMST should preferentially be applied within prospective clinical trials.**

**Recommendation 37: The chin-tuck maneuver should be used to improve swallowing safety in patients with impaired oral bolus control and consecutive premature spillage with subsequent predeglutitive aspiration.**

**Recommendation 38: Effortful swallowing can be used to improve tongue strength and swallowing physiology.**

**Recommendation 39: A systematic, regular and individualized behavioral swallowing therapy should be used early on in patients with neurogenic dysphagia, especially in patients with post-stroke dysphagia.**

Exercises and maneuvers probably constitute the most widespread treatment approach for patients with neurogenic dysphagia. In German-speaking countries, non-swallow specific concepts such as Kay Coombes’ Facial-Oral Tract Therapy (F.O.T.T.®) and Castillo Morales’ orofacial regulation therapy (ORT) are as well used as the so-called functional dysphagia therapy (FDT), which was significantly developed and put into practice by Gudrun Bartolome. The latter approach selects interventions according to the concrete pattern of neurogenic dysphagia that is present in a given patient [162, 163]. Most studies in this scientific area are devoted to the FDT or single elements of it.

Restorative techniques are intended to restore impaired swallowing functions or to promote residual functions. This is done via pre-swallow stimulation (e.g., thermal stimuli), mobilization techniques (e.g., tongue pressing against resistance), and specific motor exercises (e.g., Shaker exercise, Masako maneuver, EMST).

In contrast, compensatory methods are used during the swallow to enable effective and safe deglutition despite functional impairments. A distinction is made between postural maneuvers (e.g., chin-tuck or head-turn maneuvers) and special swallowing techniques (e.g. Mendelsohn maneuvers, supraglottic swallowing). Despite their great importance for the treatment of dysphagia in everyday care, the scientific evidence for the efficacy of this type of treatment is heterogeneous with a general lack of large RCTs providing clinical meaningful endpoints [164].

The Shaker head lift is one of the best studied exercises used in dysphagia rehabilitation for many years and is designed for patients with weakness of the suprahyoid muscles and impaired opening of the upper oesophageal sphincter [162, 163]. This procedure is a head rising exercise with an isometric high-intensity portion with three head lifts held for 60 s with a 60 s rest period between each one and an isokinetic low-intensity portion that included 30 consecutive head lifts of constant velocity without holding. The Shaker head lift has been evaluated in systematic reviews [165] and several RCT [166–169] showing that this treatment improves strengths and endurance of the suprahyoid muscles and upper oesophageal sphincter opening. In addition, there is evidence that residues and aspiration events are reduced.

The tongue muscles can also be trained through targeted exercises. Basically, tongue strength decreases with age [170], and reduced tongue strength proved to be a risk factor for aspiration [171]. Tongue strength training has been evaluated in several cohort studies and one RCT for the treatment of neurogenic dysphagia. These trials report different improvements of swallowing variables like vallecular residues and swallowing safety [172, 173].

The Masako maneuver involves swallowing while protruding the tongue beyond the lips, holding it between one’s teeth. It is meant to have a strengthening effect on the tongue and the pharyngeal walls after a period of training [162, 163]. Studies in healthy subjects did not find immediate effects on swallowing physiology [174]. A RCT including healthy subjects exposed to a four-week training with the Masako maneuver or a control task found no effect on the swallow [175]. In a small RCT recruiting subacute stroke patients the Masako maneuver was compared with neuromuscular electrical stimulation. In that trial both groups showed improvement of swallowing function, however, since a control group was missing, these results need further confirmation [176].

The so-called Lee Silverman Voice Treatment (LSVT-LOUD®) was originally developed to treat Parkinson-related dysphonia. In two smaller observational studies on 8 respectively 20 Parkinson patients, the authors also found improvements in various parameters of the oral and pharyngeal phase evaluated by VFSS [177, 178].

Expiratory muscle strength training (EMST) involves exhaling quickly and forcefully into a mouthpiece attached to a one-way valve, blocking the flow of the air until the patient produces sufficient expiratory pressure. It is meant to strengthen the expiratory and submental muscles by increasing the physiologic load [164]. This treatment has shown significant effects on swallowing safety in a RCT in Parkinson patients [179], has improved swallowing safety and feeding status in an RCT in subacute stroke patients [180, 181], has been associated with positive effect on swallowing-related muscle strength in elderly participants [182], and improved swallowing safety in patients with multiple sclerosis [183]. In ALS patients, an RCT and a cohort-study with pre-post design found that EMST improved swallow kinematics, in particular hyo-laryngeal elevation [80]. In patients with Huntington's disease, on the other hand, no effect of EMST was found on various parameters of swallowing physiology and clinical endpoints [184]. A meta-analysis summarizing this evidence across different disease categories also came to a positive conclusion [185].

The chin-down is a technique used for patients who have decreased airway protection associated with delayed swallow initiation and/or reduced tongue base retraction. To perform this maneuver, patients lower the chin towards the chest and keep this position during swallowing [162, 163]. In several studies physiological changes like expansion of the vallecular recesses, approximation of the tongue base toward the pharyngeal wall, narrowing of the entrance to the laryngeal vestibule, expedited onset of laryngeal vestibule closure, reduction in distance between hyoid and larynx, and increased duration of swallowing apnea [186]. In two well-designed cohort studies the aspiration risk could be reduced by 50% [187, 188].

In patients with unilateral pharyngeal palsy, a head turn towards the paretic side may be applied, which, if the respective swallowing impairment also affects the oral swallowing muscles, may be supplemented by a head tilt to the non-affected side [162, 163]. These maneuvers allow swallowing over the non-affected side and thus lead to a more effective pharyngeal bolus transfer [189].

The effortful swallow is mainly used in patients with an inefficient swallowing act characterized by residues in the valleculae or the sinus piriformes [162, 163]. A variety of effects on swallowing physiology could be attributed to this maneuver in studies involving both, healthy subjects and patients with neurogenic dysphagia. Thus, the effortful swallow has been shown to increase hyolaryngeal excursion, duration of hyoid elevation and UES opening, laryngeal closure, lingual pressures, peristaltic amplitudes in the distal oesophagus and pressure and duration of tongue base retraction in healthy subjects [190, 191]. In an RCT in which healthy subjects were treated with either effortful swallowing or a sham exercise, a non-significant increase in tongue strength was found in the treatment group after 4 weeks of intervention [192]. In a small RCT, in which dysphagic stroke patients either used effortful swallowing or performed a sham exercise (saliva swallowing), the intervention was associated with a significant improvement in tongue strength and oral swallowing function [193]. In addition, a small cohort study of Parkinson patients showed an increase in manometric pharyngeal pressure levels [194].

The Mendelsohn maneuver is a technique used for patients with decreased hyolaryngeal excursion and/or decreased duration of UES opening and is frequently combined with some form of biofeedback to help the patient perform it. To execute this maneuver, patients are instructed to keep the thyoid cartilage for several seconds in an elevated position before finishing the swallow [162, 163]. Studies with healthy subjects, have demonstrated with different methods of instrumental assessment that the Mendelsohn maneuver leads to various changes in the swallowing process. In particular a prolonged contraction of the submental and pharyngeal muscles, as well as the hyolaryngeal elevators have been witnessed [195]. According to a recent review, the effect on hyolaryngeal elevation can be improved by simultaneous EMG biofeedback [100]. In a small observational study, the combined use of effortful swallowing and the Mendelsohn maneuver in 3 dysphagic stroke patients reduced the aspiration risk. Long-term effects of the Mendelsohn maneuver have been evaluated in one RCT in stroke patients [196, 197]. In that study the authors could demonstrate that hyoid movement and upper oesophageal sphincter opening improved after treatment.

The super-supraglottic swallow is used as compensatory maneuver for patients with reduced airway closure. This maneuver involves the patient holding a tight breath, swallowing while keeping the airway closed, then immediately coughing after the swallow. It has been shown in several studies that the super-supraglottic swallow has immediate effects on swallowing physiology [198]. Studies with relevant clinical endpoints are not available so far [164].

Since patients with neurogenic dysphagia usually have variable and complex disorders, a combination of various adaptive, compensatory and restorative techniques has often been used in intervention studies. In their systematic review, Speyer et al. summarized 4 RCTs and 27 non-randomized studies, most of which found a significant improvement in swallowing function and other related endpoints [152]. In a Cochrane Review, updated in 2018, with focus on the treatment of post-stroke dysphagia, the use of behavioral techniques showed no effects on the key endpoints mortality and global functional outcomes. However, behavioral swallowing interventions was associated with a significant improvement in swallowing function and there was a trend for a reduction in length of hospital stay and a reduction in respiratory complications [199]. The largest RCT to date has been performed by Carnaby and co-workers [200] in stroke patients. The authors randomized 306 patients with acute dysphagic stroke to a control group receiving speech therapy according to local conditions, or in two therapy groups receiving either standardized, low-frequency or standardized high-frequency dysphagia therapy. The primary endpoint of the study was the proportion of patients taking a regular oral nutrition six months after stroke. Although the primary endpoint was narrowly missed (56% of the control group and 67% of the two therapy groups achieved the primary endpoint), standardized behavioral swallowing interventions (either low or high-intensity) showed a trend to reduce the combined endpoint of mortality or institutionalization and significantly reduced the rate of medical complications and the frequency of bronchopneumonia [200].

In addition, several comprehensive treatment programs have been evaluated in non-randomized trials. The McNeill dysphagia treatment protocol improved swallowing physiology in an observational study [201], as well as diet and clinical swallowing ability in a case-control and cohort study [202, 203]. The intensive dysphagia rehabilitation protocol was tested in a small observational study and improved the severity of aspiration and level of oral intake [204]. Similar results were found in a prospective study recruiting a small cohort of dysphagic geriatric patients. Here, swallowing function improved after 8 weeks of systematic swallowing treatment [205].

### Oral hygiene in patients with neurogenic dysphagia

**Recommendation 40: In patients with neurogenic dysphagia, good oral health should be established to reduce the risk of pneumonia and, if necessary, consistent oral hygiene should be performed.**

Poor oral health in combination with dysphagia has been identified in particular in stroke and geriatric patients as a risk factor for aspiration pneumonia [206, 207]. In addition to periodontitis, gingivitis, plaque formation and caries, respiratory pathogens such as Staphylococcus aureus, Streptococcus pneumoniae, Haemophilus influenzae, Klebsiella oxytoca, Pseudomonas aeruginosa and Escherichia coli have frequently been detected in the oral cavity of these patients [208]. The aspiration of bacterial contaminated saliva is therefore considered to be the main pathogenic mechanism of pulmonary infections in severely dysphagic patients fed via a gastric tube [209]. In order to avoid aspiration-related respiratory infections, interventions to improve oral health and reduce oral germ load are considered as therapeutic option in various collectives. Studies in stroke patients have shown that both, establishing simple protocols for the oral hygiene and also the use of more complex procedures for oral and dental cleaning, lead to an improvement of oral health [210]. In two RCTs the rate of respiratory infections in the intervention group was significantly lower than in the control group [211, 212], while another study failed to demonstrate such an effect [213]. In another RCT, the effect of selective oral decontamination was evaluated. In this study, both the pneumonia rate as well as the proportion of patients being colonized with oral pathogenetic bacteria were reduced in the intervention group that received non-absorbable antibiotics and antifungals, while there was no difference in mortality [214]. In several cohort studies, RCTs and systematic reviews targeting mixed geriatric collectives and nursing home residents different forms of oral hygiene (regular brushing of teeth, chlorhexidine mouth rinses, professional dental cleaning) also reduced pneumonia rates [215–219], while a smaller number of studies could not confirm this effect [220–222]. Consistent oral hygiene is also part of the already mentioned "Minimal-Massive Intervention (MMI)" to avoid aspiration pneumonia in frail elderly people [161].

### Pharmacotherapy of neurogenic dysphagia

**Recommendation 41: Before initiating pharmacotherapy in patients with neurogenic dysphagia, the pattern of swallowing impairment should be determined as precisely as possible.**

**Recommendation 42: Pharmacological therapies of neurogenic dysphagia can be considered as a supplement to behavioral swallowing interventions in particular in patients with a delayed swallow response.**

**Recommendation 43: Due to the limited evidence for pharmacological therapeutic approaches, these therapies should be considered on a case-by-case basis based on a careful risk-benefit analysis.**

Pharmacological treatment of OD involves the use of drugs that stimulate the neural pathways of deglutition either on the peripheral sensory level or at different levels of the central nervous system [20]. Classes of pharmacological agents that have been evaluated for their potential to improve disordered swallowing are TRPV1 agonists (Transient Receptor Potential Cation Channel Subfamiliy 1), ACE-inhibitors, dopaminergic agents and Sigma-1 receptor agonists. Currently, the potential of this treatment approach has not been fully explored. Despite some promising studies focused on swallowing physiology and a few well-made Proof-of-Principle-studies, sufficiently large multicenter RCTs with clinically relevant endpoints are not available for any of the mentioned pharmaceuticals.

TRPV1 agonists, in particular capsaicinoids and piperine, stimulate TRPV1 receptors expressed at free nerve endings of the superior laryngeal nerve and the glossopharyngeal nerve [223]. In several case-control studies, observation studies and three RCTs in different patient collectives, it has been shown that these substances increase the safety of the swallowing by shortening the latency of the swallowing reflex, by shortening laryngeal vestibule closure time and improving laryngeal elevation [71, 224–226]. In another RCT, the administration of capsaicin was associated with an increase in salivary substance P and an improvement in subjective swallowing capacity [227]. Finally, an RCT in stroke patients using capsaicin in addition to defined dietary and behavioral interventions showed that clinically evaluated swallowing function showed better recovery in patients receiving capsaicin compared to placebo after a 3-week treatment period [228]. However, studies with clinically relevant endpoints are still missing.

Disease-related loss of dopaminergic neurons, e.g. due to stroke or neurodegenerative diseases, contributes to the development of neurogenic dysphagia and is particularly associated with a delayed swallowing reflex [229]. Application of L-Dopa has been shown to normalize the onset of the pharyngeal swallow in a RCT with cross-over design that recruited patients with post-stroke dysphagia [230]. A second RCT, which also recruited chronic stroke patients, showed that nocturnal aspiration episodes could be reduced by a treatment with either amantadine or the dopamine receptor agonist cabergoline [231]. Finally, in the largest RCT to date that recruited 163 chronic stroke patients with persistent dysphagia, Nakagawa and co-workers showed that treatment with 100 mg amantadine per day significantly decreased the rate of pneumonia over the study period of three years [232].

ACE inhibitors are among the most commonly used antihypertensives. Their typical side effects include a dry cough caused by a reduced degradation of bradykinin and substance P. Substance P, which is released by free nerve endings in the pharynx and larynx, is known to enhance the swallow and cough reflex and there is evidence that decreased sputum levels of this neurotransmitter are associated with aspiration pneumonia [233]. In accordance with this pathophysiological concept, ACE inhibitors have been shown to shorten the latency of the swallowing reflex, increase the spontaneous swallowing frequency and reduce the risk of nocturnal aspiration [234–236]. Although these data suggest that ACE inhibitors can in principle lead to a strengthening of protective reflexes, studies targeting clinically relevant endpoints, in particular aspiration pneumonia, provided inconsistent results so far. On the one hand, a meta-analysis considering 5 RCTs and several case-control studies described a significant reduction in pneumonia risk associated with ACE inhibitor therapy [237]. On the other hand, a multicenter RCT randomizing tube-fed post-stroke patients to 2.5 mg Lisinopril or placebo was prematurely terminated because of an excess of mortality in the intervention group. There was no difference in the incidence of pneumonia [238].

Dextrometorphan (DM) is a weak NMDA receptor antagonist and also a Sigma-1 receptor agonist. Sigma-1 receptors are mainly found in the cerebellum and brain stem and, in particular, have been detected in bulbar motor neurons [239]. Probably by using this biochemical pathway, DM in combination with quinidine (DM/C), which inhibits its degradation, has been shown to improve pseudobulbar affect disorder in patients with ALS and MS. In 2010, DM/C was therefore approved by the FDA for this indication and in the following years the use of this drug in everyday clinical practice was extended to patients with Parkinson's disease and dementia [240]. In view of its pharmacological properties, Smith and colleagues investigated in a randomized clinical study using a cross-over design whether DM/C also had an impact on dysphagia in patients with ALS and clinically relevant bulbar symptoms [241]. As a main result, this study showed a significant improvement of subjectively perceived swallowing function (primary endpoint), while no effect on objective parameters of swallowing function were found (secondary endpoint).

### Neurostimulation

**Recommendation 44: Before initiating dysphagia treatment with a neurostimulation approach, the pattern of swallowing impairment should be determined as precisely as possible.**

**Recommendation 45: All neurostimulation methods should be used as a supplement to the behavioral swallowing therapy.**

**Recommendation 46: Due to limited data, neurostimulation methods in principle should be used in clinical trials or registries.**

**Recommendation 47: Pharyngeal electrical stimulation (PES) should be used to treat dysphagia in tracheotomized stroke patients with supratentorial lesion. Participation in prospective clinical registries is recommended.**

In recent years, various methods of peripheral (neuromuscular electrical simulation (NMES), pharyngeal electrical stimulation (PES)) and central neurostimulation (repetitive transcranial magnetic stimulation (rTMS), transcranial direct current stimulation (tDCS)) have reached a stage of development that makes their use in the clinical context outside from controlled trials increasingly conceivable in the near future [26]. Although these methods have been tested in a number of studies and in different patient populations of late, larger multicenter RCTs with clinically relevant endpoints are needed for a final assessment of their respective effectiveness.

Neuromuscular electrical simulation (NMES) stimulates sensory or motor nerve fibers involved in swallowing transcutaneously. Its mechanism of action is thought to include accelerating the development of muscle strength and promoting central nervous system recovery. NMES is commonly used in addition to behavioral swallowing therapy. Meta-analyses of predominantly smaller randomized and non-randomized studies showed a moderate effect of NMES on swallowing function and level of diet [242–245]. These findings have been confirmed in two RCTs. Park and co-workers showed improved hyoid-movement in subacute stroke patients after treatment with NMES in combination with effortful swallowing compared to effortful swallowing alone [246]. Terre and Mearin found improved feeding status in patients with OD after stroke or traumatic brain injury when being exposed to NMES and conventional swallowing therapy compared to conventional swallowing therapy alone [247]. Another scientifically sound RCT, however, showed no additional benefit of sensory or motor NMES when supplementing behavioral swallowing therapy in patients with Parkinson's disease related dysphagia. Regardless of whether the patients received behavioral swallowing therapy alone or combined with sensory or motor NMES, a similar improvement of a variety of oral and pharyngeal phase parameters were demonstrated by instrumental swallowing assessment [248].

In pharyngeal electrical stimulation (PES), the tongue base and the back of the pharyngeal wall are electrically stimulated via a transnasally inserted feeding tube housing a pair of bipolar ring electrodes. PES induces neuroplasticity within the swallowing network by targeting the pharyngeal motor and sensory cortices and possibly also working on the peripheral sensory afferent system. Muscle contraction, in contrast to NMES focusing on pure muscle strengthening, is not the aim of treatment. In smaller RCTs in dysphagic stroke patients and patients with neurogenic dysphagia due to multiple sclerosis, PES has been shown to improve dysphagia and, in some cases, even shorten the times to hospital discharge [249–251]. The STEPS study, however, a large multicenter RCT investigating the effect of PES for the treatment of dysphagia in acute and subacute stroke patients, showed no effect of the intervention compared to sham stimulation [252]. On the other hand, the multi-center PHAST-TRAC study, which recruited severely dysphagic, tracheotomized stroke patients with supratentorial lesions, showed a significant benefit of PES. While in the therapy group almost 50% of patients could be decannulated after a three-day PES intervention, in the control group a spontaneous remission of dysphagia allowing the patients to be decannulated, was witnessed in only 9% of the patients [126]. A meta-analysis that considered the results of a single center RCT in addition to PHAST-TRAC [253], confirmed this therapeutic effect [126].

Both rTMS (repetitive transcranial magnetic stimulation) and tDCS (transcranial direct current stimulation) have been used for direct, non-invasive stimulation of the swallowing network with the aim of influencing the functionally relevant level of excitability and activity [254]. In the meantime, a large number of smaller RCTs and cohort studies were summarized in several meta-analyses, which were able to show a moderate but persistent therapeutic effect on swallowing function for both neurostimulation methods [245, 255–257]. In the largest single-center RCT on this topic to date, contralesional tDCS in acute dysphagic stroke patients was not only associated with an improvement in dysphagia, but also a neurophysiological detectable modulation of the swallowing network was found in spatial proximity to stimulation [258]. Apart from post-stroke dysphagia, a positive effect of transcranial stimulation on swallowing function was also demonstrated in MS patients with strategic brain stem lesion [259, 260]. In addition to the supratentorial stimulation evaluated in these studies, there is also initial evidence that cerebellar stimulation can also contribute to a reorganization of the swallowing network and improve swallowing function [261, 262].

### Treatment of hypersalivation in patients with neurogenic dysphagia

**Recommendation 48: Debilitating hypersalivation in patients with neurogenic dysphagia can be treated with botulinum toxin injections into the salivary glands and/or anticholinergic drugs.**

**Recommendation 49: If pharmacological treatment does not provide adequate symptom control or if side effects prevent from its continuation, radiotherapy of the salivary glands may be considered.**

The treatment of hypersalivation, a condition which is highly important for the management of patients with neurogenic dysphagia, has been comprehensively elaborated in the S2k guidelines “Hypersalivation”. This guideline was developed by the German Society of Otolaryngology, Head and Neck Surgery (DGHNO KHC) with the participation of other professional societies and associations (DGPP, DGSS, DGPPN, DGN, DGP, DPV, DNP, DEGRO, DGMKG) and was updated in 2019 [27]. Therefore, a comprehensive presentation of this topic is omitted here and the available therapeutic options are summarized referring to the guideline above.

The pharmacological therapy of hypersalivation consists on the one hand in the inhibition of the salivary glands by anticholinergic muscarin-receptor antagonists. These agents can be administered orally, intravenously, by intramuscular injection, transdermally or quasi local (e.g. sublingual application of drops or spray). In Germany, atropine, scopolamine, and glycopyrrolate are mainly used. The application of these substances in adults is off-label, only glycopyrrolate has been approved for the symptomatic treatment of severe hypersalivation in children from 3 years on and adolescents throughout Europe in 2016. On the other hand, the cholinergic neuroglandular transmission of the salivary glands can be reversible and significantly reduced by the intraglandular injection of botulinum toxin into the large salivary glands. After successful completion of an RCT in which 184 patients with typical (70.7%) or atypical (8.7%) Parkinson syndromes, stroke (19%) or traumatic brain injury (2.7%) were included, Incobotulinum toxin A has been approved in Europe for the treatment of hypersalivation in adults irrespective of the underlying etiology in 2019 [263, 264]. Radiotherapy can also be used for the treatment of hypersalivation in individual cases, e.g. if the treatment with anticholinergic drugs or the injection therapy with botulinum toxin do not provide sufficient symptom control or repeated injections are not feasible. While the fundamental and long-lasting efficacy of external irradiation of the salivary glands has been convincingly demonstrated, the possible side effects as well as the inherent carcinogenic potential must be taken into account [27].

### Minimally invasive and surgical therapies

**Recommendation 50: For the treatment of cricopharyngeal dysfunction with impaired opening of the UES, cricopharyngeal myotomy (open or endoscopic), dilatation (by balloon or bougie) and botulinum toxin injection (transcutaneous or endoscopic) are considered.**

**Recommendation 51: The indication should be made by a multi-professional team of experts. The procedure should only be carried out at specialized centers.**

**Recommendation 52: The indication for interventional or surgical treatment of cricopharyngeal dysfunction and impaired opening disorder of the UES in the context of neurogenic dysphagia should consider the following criteria:**

1. **The diagnosis is based on VFSS and HRM.**
2. **The phenotype and etiology of dysphagia have been clarified.**
3. **A sufficiently long (approx. 1 year) conservative therapy (treatment of the underlying disease; swallowing therapy by Shaker exercise, Mendelsohn maneuvers, EMST) has not been successful.**
4. **A refractory gastro-oesophageal reflux has been ruled out.**
5. **A sufficient hyolaryngeal elevation is present.**

**Recommendation 53: For the treatment of therapy-refractory glottal closure insufficiency, minimal-invasive surgical procedures for medialization of the vocal folds may be chosen. This treatment aims at improving cough and reducing the risk of aspiration.**

Minimal-invasive and surgical therapy procedures can be applied for the treatment of severe opening disorders of the UES, if this disorder is relevant to the overall impression of the swallowing disorder. In case of cricopharyngeal myotomy the muscles forming the UES (cricopharyngeal muscle, inferior pharyngeal constrictor muscle as well as the upper striated muscles of the oesophagus) either can be cut through in the longitudinal direction by open or endoscopic access [92, 265]. Minimal-invasive treatment options consist of the dilatation of the UES (with a balloon or bougie) [266] and the endoscopic or transcutaneous injection of botulinum toxin [267]. These procedures have so far been tested in patients with inclusion body myositis, oculopharyngeal muscle dystrophy, multiple sclerosis, amyotrophic lateral sclerosis, stroke and M. Parkinson. As consistently summarized in several reviews and two Cochrane analyses, these therapeutic options have mainly been evaluated within retrospective, uncontrolled case series [92, 265, 268]. Only in one randomized pilot study balloon dilatation was compared with laser myotomy in 8 patients. Both treatments resulted in an increase in the diameter of the UES and a subjective improvement of dysphagia [269]. In a systematic review summarizing the results of 32 studies, available therapeutic options were compared in terms of effectiveness and side effects [270]. Weighted average success rates of each intervention were 78% for myotomy (84% for endoscopic myotomy and 71% for open surgery), 73% for dilatation and 49% for botulinum toxin injection. The weighted average complication rates were 7% for myotomy (2% for endoscopic myotomy and 11% for open surgery), 5% for dilatation and 4% for botulinum toxin injection. Complications included fistula, supraglottic edema, mediastinitis, retropharyngeal hematoma, oesophageal injuries, laryngospasm and severe bleeding [270]. Although these figures do not allow for a reliable comparison of the different methods due to limited data, they nevertheless indicate that all of the therapeutic options basically may be employed. Since the indication is difficult and side effects can be serious, even life-threatening, these interventions should only be performed in specialized centers involving surgeons, gastroenterologists, otolaryngologists and neurologists with appropriate relevant expertise. The following expert recommendations can be considered in this context. 1. Any intervention targeting the UES should be performed after comprehensive diagnostics including VFSS and HRM; 2. An etiological classification of the underlying dysphagia should have been made; 3. A sufficiently long (approx. 1 year) conservative therapy (treatment of the underlying disease; swallowing therapy by Shaker exercise, Mendelsohn maneuvers, EMST) should have been carried out and found to be ineffective; 4. The presence of a therapy-refractory reflux should have been ruled out; 5. A sufficient hyolaryngeal elevation should be present.

Minimally invasive and surgical procedures are also helpful for the treatment of glottal insufficiency (GI). In addition to improving the quality of voice, these treatments may also lead to an improvement in swallowing safety. Vocal fold palsy due to the affection of the vagal nerve and its branches as well as pronounced vocal fold atrophies, which occur e.g. in M. Parkinson or ALS [271], can cause GI that causes an impairment of laryngeal protective functions [272]. Aspiration and reduced cough strength with subsequent impaired laryngeal and bronchial clearing are likely consequences [273]. In the case of brain stem or vagal nerve lesions, the risk of aspiration is particularly high due to the associated UES dysfunction and severe sensory loss [272].

If, despite sufficient long and intensive speech therapy, no sufficient improvement of GI is achieved, surgical medialization techniques can be used as support. As a consequence of medializing the vocal fold, the laryngeal protective function improves by preventing aspiration and increasing cough strength [274]. The treatment strategy of a persistent GI with aspiration essentially comprises of vocal fold augmentation, which is performed in local anaesthesia and office-based with temporary or permanent injectates [275, 276]. In case of larger GIs, thyroplasty, in which the vocal fold is medialized from the outside through the laryngeal framework, e.g. with a silicone wedge, goretex or titanium, has been established [277].

Although glottic narrowing interventions have been successfully used for many years to improve voice and swallowing, there are no randomized trials or systematic reviews investigating their effectiveness in neurogenic dysphagia. However, the results of the published case series and smaller case studies mostly show that the medialization techniques improve not only the subjective but also the objective swallowing capacity by reducing aspiration and improving the cough [278, 279]. In vocal fold augmentation, the complications are generally rare and mainly include laryngeal edema, material intolerance, overcorrections and bleeding. Since with the help of laryngoscopy indicating this treatment is simple and the procedure is gentle and quick, the medialization of the vocal fold can be a useful supplement in the therapy of neurogenic dysphagia with GI.

## Data Availability

Not applicable.
